# Timing malaria transmission with mosquito fluctuations

**DOI:** 10.1002/evl3.61

**Published:** 2018-06-22

**Authors:** Romain Pigeault, Quentin Caudron, Antoine Nicot, Ana Rivero, Sylvain Gandon

**Affiliations:** ^1^ MIVEGEC (UMR CNRS 5290) University of Montpellier Montpellier France; ^2^ Department of Ecology and Evolution University of Lausanne Lausanne Switzerland; ^3^ Princeton University Princeton NJ 08544; ^4^ CEFE (UMR CNRS 5175) University of Montpellier Montpellier France

**Keywords:** cinderella hypothesis, circadian rhythm, Hawking hypothesis, periodicity

## Abstract

Temporal variations in the activity of arthropod vectors can dramatically affect the epidemiology and evolution of vector‐borne pathogens. Here, we explore the “Hawking hypothesis”, which states that these pathogens may evolve the ability to time investment in transmission to match the activity of their vectors. First, we use a theoretical model to identify the conditions promoting the evolution of time‐varying transmission strategies in pathogens. Second, we experimentally test the “Hawking hypothesis” by monitoring the within‐host dynamics of *Plasmodium relictum* throughout the acute and the chronic phases of the bird infection. We detect a periodic increase of parasitemia and mosquito infection in the late afternoon that coincides with an increase in the biting activity of its natural vector. We also detect a positive effect of mosquito bites on *Plasmodium* replication in the birds both in the acute and in the chronic phases of the infection. This study highlights that *Plasmodium* parasites use two different strategies to increase the match between transmission potential and vector availability. We discuss the adaptive nature of these unconditional and plastic transmission strategies with respect to the time scale and the predictability of the fluctuations in the activity of the vector.

**Impact Summary**

Seasonal and daily fluctuations in the environment affect the abundance and the activity of vectors and may therefore have profound consequences on the transmission of infectious diseases. Here we show that, in accord with evolutionary theory, malaria parasites have evolved two different and complementary strategies to cope with fluctuations in mosquito availability. First, *Plasmodium relictum* adopts an unconditional strategy whereby within‐host parasitemia and mosquito infection increases in the evening, when its vector, the *Culex pipiens* mosquito, is most active. Second, we find evidence for a plastic strategy allowing the parasitemia to rapidly increase after exposure to mosquito bites.

All organisms face periodic changes in their environment. These environmental fluctuations, which can happen at time scales ranging from daily to annual, affect the physiological, immunological, and behavioral activities of all species (Smaaland et al. 2002; Corder et al. 2016; Duboscq et al. 2016) including parasites (Martinez‐Bakker & Helm 2015; Thaiss et al. 2015; Rijo‐Ferreira et al. 2017a). Both short‐term (circadian) and long‐term (seasonal) fluctuations in the environment may trigger dramatic perturbations of the physiology of the hosts that can affect the within‐host dynamics of the parasite and, ultimately, its epidemiology. One potential explanation for these parasite fluctuations is that they are a by‐product of the biological rhythms imposed by the host. There is, for example, abundant evidence of the existence of short‐term (circadian) rhythms in the expression of physiological and immune host genes that may potentially impact the development of the parasites within (Edgar et al. 2016, Prior et al. 2018). Longer term (seasonal) fluctuations may also trigger dramatic perturbations of the physiology and immunology of the host, which may affect the within‐host dynamics of some parasites (see Martinez‐Bakker & Helm 2015).

Alternatively, and arguably more interestingly, these periodic fluctuations may be viewed as pathogen adaptations aimed at maximizing transmission by taking advantage of a transient favorable environment (Hawking 1975; Martinez‐Bakker & Helm 2015). For instance, in the coccidian parasite *Isospora sp*, the highly synchronized production of transmissible stages in the feces of infected animals takes place in the late afternoon to minimize mortality through desiccation and UV radiation (Martinaud et al. 2009). Crucially, Hawking (Hawking 1970, 1975) argued that similar processes may be acting in vector‐borne diseases. He postulated that the timing and the rhythm of many vector‐borne pathogens may have evolved to match the daily fluctuations in vector abundance. This so‐called “Hawking hypothesis” (Garnham & Powers 1974; Gautret & Motard 1999) has received considerable empirical support from both within and cross‐species comparisons of microfilarial parasites, where parasite and mosquito daily rhythms seem to be well matched. For example, the parasite *Wuchereria bancrofti*, which is transmitted by night‐biting *Culex sp* mosquitoes, shows a marked nocturnal periodicity where the transmissible microfilaria are sequestered in the lungs during daytime and released into the peripheral blood at night (Hawking 1975). However, in the Pacific islands, where the parasite is transmitted by day‐biting *Aedes polinesiensis* mosquitoes, *Wuchereria bancrofti* microfilaria are significantly more abundant during the day (Moulia‐Pelat et al. 1993).

Many malaria parasites exhibit striking periodic and synchronized cell cycles leading to the simultaneous burst of infected red blood cells at regular points in time. In spite of numerous studies exploring the adaptive nature of malaria periodicity in relation to vector activity (Hawking 1970, 1975; Gautret & Motard 1999) whether these patterns fit the “Hawking hypothesis” remains a controversial issue. For instance, Mideo et al. (2013) questioned the ability of malaria parasites to coordinate gametocyte production and maturation to reach maximum infectiousness when mosquitoes feed. Gametocyte maturation (the process under which gametocytes become infective to mosquitoes; Alano 2007) may, however, be decoupled from the rest of the parasite's life cycle. Although the process of gametocyte maturation is still not well understood, potential inducers of gametocyte maturation have been described (Sinden 2015), some of which may be under circadian control (Rijo‐Ferreira et al. 2017b). Also, Hawking (1966) observed that “the cycle of infectivity is not due to the cycle of the number of gametocytes in the blood but must be due to variation in their physiological state—i.e., their suitability to develop in mosquitoes”. This suggests that the periodic fluctuation of malaria infectivity is not driven solely by gametocyte abundance. One of the main objections against the “Hawking hypothesis” is, however, the lack of evidence for a match between the parasite's cycles in infectivity and the biting activity of mosquitoes (Mideo et al. 2013). Indeed, the large majority of studies aiming to test this hypothesis in malaria have focused on the within‐host dynamics of the parasite, without testing whether this translates into higher mosquito infection.

Here, we first present a theoretical model that studies evolution of time‐varying transmission strategies of *Plasmodium* in a periodically fluctuating environment. This model identifies the conditions under which a periodic investment in transmission is expected to evolve. Then, we carry out an experiment to explore empirically the validity of the “Hawking hypothesis”. For this purpose, we study the periodicity of the avian malaria parasite, *Plasmodium relictum*, in relation with the timing of the activity of its natural vector in the field, the mosquito *Culex pipiens*. In contrast with human malaria, *P. relictum* does not exhibit synchronous development in its vertebrate host (all erythrocytic stages are present in the blood at all times) but several earlier studies report daily fluctuations in within‐host parasite abundance (see Gambrell 1937; Hewitt 1940). Yet, the potential link between these fluctuations and the activity of the mosquito vectors remains to be investigated. To explore the validity of the “Hawking hypothesis”, we monitored both blood parasitemia (Pigeault et al. 2015) and mosquito activity throughout the day. We used overall parasitemia as a proxy for transmissible stage (gametocyte) production because in avian malaria the development of gametocytes follows quite closely the development of asexual forms (Hewitt 1940) and our previous work (Pigeault et al 2015) has shown that there is a very good correlation between sexual (gametocyte) and asexual parasitemia. As pointed out by our theoretical analysis, the adaptive scenario underlying the “Hawking hypothesis” should yield a positive covariance between bird parasitemia and mosquito activity.

We worked on both the acute and chronic stages of the infection. From the point of view of the parasite, these two stages are fundamentally different in terms of transmission opportunities. While the acute phase is very short‐lived and results in high rates of mosquito infection, the chronic phase can last several months, and even years, but does not yield high transmission rates (Cornet et al. 2014; Pigeault et al. 2015). We thus compare these two phases of the infections to establish: (1) the existence of fluctuations of blood parasitemia throughout the day and (2) whether these fluctuations translate into higher pathogen transmission to mosquitoes. To explore the periodicity in host parasite density, we applied a classical statistical approach in the analysis of circadian rhythms that allows to correct the data for non‐stationarities in the host parasitaemia caused by the large‐scale changes in within‐host dynamics during the acute phase of the infection (Ruf 1999, Deckard et al. 2013). In addition, given that mosquito bites may themselves affect within‐host dynamics of the parasite (Lawaly et al. 2012; Cornet et al. 2014; Reece & Mideo 2014) we compared the within‐host dynamics of malaria in birds exposed (or not) to mosquitoes. Indeed, mosquito bites may be another signal the pathogen may use to respond to the variability of the availability of the vector, albeit at a different (shorter) temporal scale. We have previously argued that such a strategy may be an adaptation to a fluctuating seasonal environment where mosquitoes are very abundant during certain seasons and absent during others (Cornet et al. 2014; Reece & Mideo 2014). The present paper is an attempt to explore another dimension of malaria adaptation to fluctuations in mosquito availability. In the following, we show that *Plasmodium* parasites can use both constitutive and plastic variations in within‐host investment in transmission to match short‐term and long‐term fluctuations in vector availability.

## Material & Methods

### THEORETICAL MODEL: EVOLUTION OF ADAPTIVE RHYTHMICITY

To explore the evolution of rhythmic transmission strategies, we use a simple epidemiological model of malaria. The vertebrate host population is assumed to be constant and equal to $N_{H}=S\left(t\right)+I\left(t\right)$, where S(t) and I(t) are the densities of uninfected and infected hosts, respectively. Similarly, the mosquito vector population is also assumed to be constant and equal to $N_{V}=V\left(t\right)+V_{I}\left(t\right)$, where V(t) and $V_{I}\left(t\right)$ are the densities of uninfected and infected vectors, respectively. The activity of the vector a(t) is assumed to fluctuate with a period T=1 day. Low mosquito activity decreases biting rate and transmission and, consequently, the epidemiological dynamics fluctuate periodically. The following set of differential equations describes the temporal dynamics of the different types of hosts (the dot notation indicates differential over time):

(1) $$\begin{array}{c} \dot{I}\left(t\right)=\left(N_{H}-I\left(t\right)\right)V_{I}\left(t\right)a\left(t\right)\beta_{2}-\left(d+\alpha \left(t\right)\right)I\left(t\right)\\ {\dot{V}}_{I}\left(t\right)=I\left(t\right)\left(N_{V}-V_{I}\left(t\right)\right)a\left(t\right)\beta_{1}\left(t\right)-m_{I}V_{I}\left(t\right) \end{array}$$

where *d* is the natural mortality rate of the vertebrate host and α is the virulence of malaria (the extra mortality induced by the infection); $m_{I}$ is the mortality rates of infected vectors; $\beta_{1}\left(t\right)$ is the transmission rate from the vertebrate host to the vector; and β_2_ is the transmission rate from the vector to the vertebrate host. Note that the pathogen is allowed to have time‐varying investment in transmission, $\beta_{1}\left(t\right)$, and virulence, α(t), in the vertebrate host. In this system investment in transmission and virulence are strongly correlated (Pigeault et al 2015). Therefore, as in classical models of virulence evolution, replication allows the parasite to transmit more efficiently (i.e., higher $\beta_{1}\left(t\right)$) but is assumed to be costly because it may induce the death of the vertebrate host (i.e., higher α(t)). For the sake of simplicity, this epidemiological model lacks several classical features of the biology of malaria transmission (e.g., no extrinsic incubation period in the mosquito, no explicit description of within‐host dynamics in the vertebrate host). Unlike other models that analyzed the optimal investment in transmission of malaria parasites (Greischar et al. 2016), this model retains a key element involved in the rational of the “Hawking hypothesis”: a time‐varying activity of the vector.

To study parasite evolution, we track the dynamics of a rare mutant parasite *M* with different transmission and virulence strategies ($\beta_{1M}\left(t\right)$ and $\alpha_{M}\left(t\right)$, respectively:

(2) $$\begin{array}{c} {\dot{I}}_{M}\left(t\right)=\left(N_{H}-I\left(t\right)\right)V_{\mathrm{IM}}\left(t\right)a\left(t\right)\beta_{2}-\left(d+\alpha_{M}\left(t\right)\right)\;I_{M}\left(t\right)\\ {\dot{V}}_{\mathrm{IM}}\left(t\right)=I_{M}\left(t\right)\left(N_{V}-V_{I}\left(t\right)\right)a\left(t\right)\beta_{1M}\left(t\right)-m_{I}\;V_{\mathrm{IM}}\left(t\right) \end{array}$$

Because the frequency of the fluctuation in mosquito activity is much higher than other dynamical variations of the system, we may assume that the density of infected hosts remains approximately stable throughout the day. This separation of time scale allows to focus on the dynamics of the vector compartment which yields:

(3) $$\begin{array}{ccc} V_{I}\left(t\right) & \approx & \left(\frac{a\left(t\right)N_{H}\beta_{2}\left(N_{V}-V_{I}\left(t\right)\right)}{\left(d+\alpha \left(t\right)+a\left(t\right)V_{I}\left(t\right)\beta_{2}\right)}a\left(t\right)\beta_{1}-m_{I}\right)V_{I}\left(t\right)\\ {\dot{V}}_{\mathrm{IM}}\left(t\right) & \approx & \left(\frac{a\left(t\right)N_{H}\beta_{2}\left(N_{V}-V_{I}\left(t\right)\right)\left(d+\alpha \left(t\right)\right)}{\left(d+\alpha \left(t\right)+a\left(t\right)V_{I}\left(t\right)\beta_{2}\right)\left(d+\alpha_{M}\left(t\right)\right)}a\left(t\right)\beta_{1M}\left(i\right)-m_{I}\right)V_{\mathrm{IM}}\left(t\right) \end{array}$$

The change in frequency of the mutant is thus given by:

$${\dot{p}}_{M}\left(t\right)\propto A\left(t\right)\left(B_{1M}\left(t\right)-B_{1}\left(t\right)\right)\;p_{M}\left(t\right)$$

(4) With A(t)=a(t)a(t)NHβ2(NV−VI(t))(d+α(t)+a(t)VI(t)β2)B1M(t)=β1M(t)(d+αM(t)), and B1(t)=β1(t)(d+α(t))

The ability of the mutant to invade the resident population is determined by $s_{M}$, the selection coefficient on the mutant, which can be evaluated after integrating the change of the mutant frequency over 1 day:

(5a) $$s_{M}=\frac{1}{T}\int_{0}^{T}\left({\dot{p}}_{M}\left(t\right)/p_{M}\left(t\right)\right)\;\;dt,$$

which yields:

(5b) sM∝A∼(B∼1M−B∼1)︸ Classical  transmission − virulence  tradeoff +covt(A,B1M)−covt(A,B1)︸ Match  between  mosquito  activity  and  within − host  investment  in  transmission ,

where the tilde refers to the average over a period T=1 day of the fluctuation. The first term in the above equation for $s_{M}$ is akin to the classical tradeoff between transmission β_1_ and virulence α. The second term measures the benefit associated with a closer match between parasite dynamics in the vertebrate host and the rhythmicity in mosquito behavior.

## Experiment: “Hawking Hypothesis” in Avian Malaria

### MALARIA PARASITES AND MOSQUITOES

*Plasmodium relictum* (lineage SGS1) is the etiological agent of the most prevalent form of avian malaria which is commonly found infecting passeriform birds in Europe (Pigeault et al. 2015). Our parasite lineage (SGS1) was isolated from an infected house sparrow caught in the region of Saintes Maries‐de‐la‐Mer (France) in May 2015 and transferred to naïve canaries (*Serinus canaria*, Passeriforms).

Mosquito experiments were conducted with a laboratory isogenic strain of *Cx. pipiens* mosquitoes. The susceptibility to infection by *P. relictum* and the behavioral activity of our mosquito strain are similar to what is observed in wild *Cx. pipiens* mosquitoes (Vézilier et al. 2010, Pigeault pers. obs.). Mosquitoes were reared as described by Vézilier et al. (2010). We used females 7 ± 2 days after emergence that had no prior access to blood and which were starved for 6 h before the experiment. Mosquitoes and canaries were maintained under a 12:12‐h LD cycle (6 h light on, 18 h light off).

### EXPERIMENTAL DESIGN

**Figure 1:**
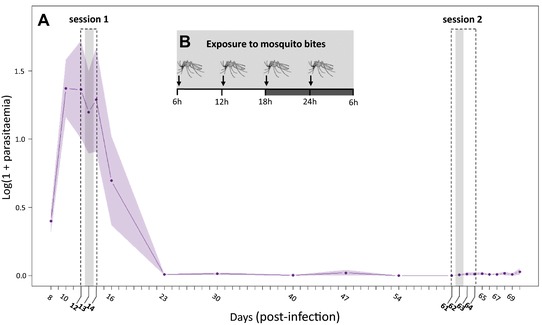
Overview of the experiment. (A) Mean bird parasitemia (Log(1 + parasitemia)) through time (includes three birds exposed to mosquito bites and four unexposed birds). Parasitemia was measured at noon using blood smear counts. The shaded envelop around the lines represents the standard error of the mean. The dashed boxes represent the two experimental sessions performed during the acute (12–14 day post‐infection) and chronic (61–64 day post‐infection) stages of the infection. In each session, the grey vertical bar corresponds to the day at which birds from the “exposed” treatment were exposed to mosquito bites (day 13 and day 62 post‐infection for the acute and chronic stages of the infection, respectively). The protocol followed on the exposure day is shown in panel **(B)** where the dark grey area (18:00–06:00 h) represents the night period. Arrows indicate the time of day at which birds were exposed to mosquito bites. Mosquito exposure was carried out straight after each of the blood sampling events (at 06:00, 12:00, 18:00, and 00:00 h). The different parasite dynamics in “exposed” and “unexposed” birds is shown in Figure 3.

Experiments were carried out between May and July 2015 using (1‐year old) domestic canaries (*Serinus canaria*). Prior to the experiments, a small amount of blood (3–5 μL) was collected from the medial metatarsal vein of each of the birds and used to verify that they were free from any previous hemosporidian infections. Eight canaries were experimentally inoculated by means of an intraperitoneal injection of ∼80 μL of an infected blood pool (day 0; Fig. 1; Pigeault et al. 2015). The blood pool was constituted of a mixture of blood from three infected canaries inoculated with the parasite isolated from the field 3 weeks before the experiment. The eight infected birds were assigned to two treatments: “exposed” (*n* = 3) or “unexposed” (*n* = 5) to mosquito bites. One “unexposed” bird lost the malaria infection very quickly (10 days post infection [dpi]) and was removed from the analyses. From day 8 to day 70 postinfection parasitemia of each bird was monitored regularly at noon (12 h, Fig. 1) except during the experimental sessions when sampling was increased to four times per day (see below for details). All blood samples were carried out by collecting 5–10 μL of blood from the medial metatarsal vein. A drop of this blood sample was smeared onto a slide for the visual quantification of the parasitemia (Valkiunas 2004), and the rest was frozen for the molecular quantification of the parasitemia (see below). In *Plasmodium relictum* infections parasitemia and gametocytemia are strongly positively correlated (see Fig. 2 in Pigeault et al. 2015). For practical reasons, parasitemia, which is more rapidly quantified, was therefore used as a proxy of parasite investment in the production of transmissible stage.

**Figure 2:**
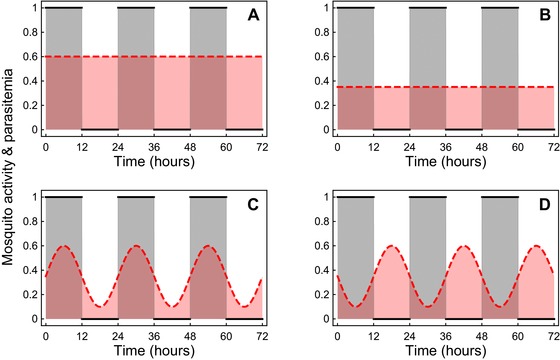
Parasite investment in transmission in a fluctuating environment. This figure illustrates four different strategies (A–D) used by parasites in an environment characterized by periodic fluctuations in vector activity (i.e., biting rate). The red area shows the parasitemia in the vertebrate host and the grey area shows mosquito activity (0 = inactive, 1 = active). Panels A and B depict two constant strategies with different investment in transmission, which may incur different fitness costs (e.g., virulence). Panels C and D depict two different time‐varying strategies (see Results section for details). Areas of overlap between the gray and red areas (dark red) represent the effective transmission rate of the parasite. Effective transmission can be maximized in a time varying strategy if there is a positive covariance between parasitemia and mosquito activity (strategy C).

#### Daily fluctuations of Plasmodium infection

In order to investigate the daily fluctuation of the blood parasitemia, two experimental sessions were carried out: the first one during the acute stage of infection (Session 1: between day 12 and 14 dpi, Fig. 1) and the second one during the chronic stage of infection (Session 2: between day 61 and 64 dpi, Fig. 1). During these two experimental sessions blood sampling was carried out every 6 h (at 06:00, 12:00, 18:00, and 00:00 h, Fig. 1B). In the acute stage of the infection, the existence of a daily fluctuation in the blood parasitemia was investigated by counting the number of parasites in blood smears (Valkiunas 2004) while in the chronic stage, when parasites in the blood were so scarce that blood smear counts were highly inaccurate, parasite intensities were calculated using molecular tools (see below). In the acute stage of the infection, the within‐day fluctuations of parasitemia may be masked by the large‐scale (between‐day) changes in parasitemia. Therefore, the periodicity of the fluctuations in bird parasitemia was analyzed using a statistical approach that takes into account the overall within‐host dynamics of *Plasmodium* infection (see Supporting Information Materials, **S1 Text**).

#### Daily fluctuations of Plasmodium transmission

In order to estimate whether fluctuations in blood parasitemia translate into fluctuations in transmission to mosquitoes we: (1) obtained estimates of mosquito activity throughout the day, (2) estimated the number of parasites ingested by the mosquitoes at different times during the day, and (3) estimated the success of the infection at the oocyst (midgut) stage. For this purpose, on day 13 (Session 1) and day 62 dpi (Session 2), and straight after each of the blood sampling events (at 06:00, 12:00, 18:00, and 00:00 h), the birds from the “exposed” treatment were placed inside a cage (L40 x W30 x H30 cm) with a batch of 70 uninfected female mosquitoes for 135 min. The remaining (“unexposed”) birds were kept under identical conditions but without the mosquitoes. The cages were visited every 45 min and all blood fed females were removed and counted. A red lamp was used to capture blood fed mosquitoes during the night (18:00 and 00:00 h) without disturbing the birds and the mosquitoes. The number of mosquitoes fed at each time step was recorded and was used an as estimate mosquito activity throughout the day (see below). Thereafter, these recently blood‐fed mosquitoes were divided in two groups. One half was frozen individually in order to quantify the parasites ingested in the blood meal (see below). The other half was kept alive to obtain an estimate of the blood meal size and of the success of the infection (number of oocysts in the midgut). This was done by placing these mosquitoes in numbered plastic tubes (30 mL) covered with a mesh with a cotton pad soaked in a 10% glucose solution. After 7 days (day 7 post blood meal), the females were taken out of the tubes and the amount of hematin excreted at the bottom of each tube was quantified as an estimate of the blood meal size (Vézilier et al. 2010). Females were then dissected and the number of *Plasmodium* oocysts in their midguts counted with the aid of a binocular microscope (Vézilier et al. 2010).

At the end of the mosquito exposure session, the parasitemia of the birds was monitored on a daily basis for a total of 57 days in acute and 8 days in chronic stage of infection. This allowed us to contrast the within‐host dynamics of the malaria parasites in birds exposed or not to mosquitoes.

### MOLECULAR ANALYSES

The molecular quantification of parasites in the mosquito blood meal was carried out using a quantitative PCR (qPCR) protocol adapted from (Cornet et al. 2013). Briefly, DNA from blood‐fed females was extracted using standard protocols (Qiagen DNeasy 96 blood and tissue kit). For each individual, we conducted two qPCRs in the same run: one targeting the nuclear 18s rDNA gene of *Plasmodium* (Primers: 18sPlasm7 5’‐AGCCTGAGAAATAGCTACC‐ ACATCTA‐3’, and 18sPlasm8 5’‐TGTTATTTCTTGTCACTACCTCTC‐ TTCTTT‐3’), and the other targeting the 18s rDNA gene of the bird (18sAv7 5’ GAAACTCGCAATGGCTCATTAAATC‐3’, and 18sAv8 5’‐TATTAGCTCTAGAATTACCACAGT TATCCA‐3’). All samples were run in triplicate (ABI 7900HT real‐time PCR system, Applied Biosystems) and their mean was used to calculate the threshold Ct value (the number of PCR cycles at which fluorescence is first detected, which is inversely correlated with the initial amount of DNA in a sample) using the software Light Cycler 480 (Roche). Parasite intensities were calculated as relative quantification values (RQ). RQ can be interpreted as the fold‐amount of target gene (*Plasmodium* 18s rDNA) with respect to the amount of the reference gene (Bird18s rDNA) and are calculated as 2 ^−(Ct18s Plasmodium – Ct18s Bird)^. For convenience, RQ values were standardized by ×10^4^ factor and log‐transformed (Cornet et al. 2013).

### STATISTICAL ANALYSIS

The statistical analyses were run using the R software (V. 3.3.3). The different statistical models built to analyze the data are described in the Supporting Information (**Table S1**). Analyses in which a same individual bird was sampled repeatedly, such as the daily fluctuation of blood parasitemia or the impact of mosquito exposure on the parasite replication rate, were analyzed fitting bird as a random factor into the models (to account for the temporal pseudoreplication), using a mixed model procedure (*lme*, package: nlme). Similarly, mosquito‐centered traits (such as infection prevalence or oocyst burden), which may depend on which bird mosquitoes fed on, were also analyzed, fitting bird as a random factor into the models (to account for the spatial pseudoreplication), using *lme* or *glmer* (package: lme4) according to whether the errors were normally (oocyst burden) or binomially (prevalence) distributed. Time of day and, when necessary, blood meal size (hematin) were used as fixed factors.

The impact of time of day on mosquito activity (i.e., time required to take a blood meal) was analyzed using survival analyses for censored survival data (survreg model, package: survival). In these analyses, we used the status “blood fed” instead of “dead” status. For this purpose, during the blood feeding period in both the acute and chronic sessions, the number of fed mosquitoes was recorded every 45 min for a period of 135 min (see above). In order to take into account that some of the mosquitoes had not fed at the end of the experiment, we added a censoring indicator vector. Time of day and experimental session were fitted as fixed factors in a parametric model with exponential distribution. Constant hazard rates λ were obtained from this model as an estimate of the speed at which mosquitoes take a blood meal at different times of day (06:00, 12:00, 18:00, and 00:00 h). A higher hazard rate equates to a higher mosquito activity.

Maximal models, including all higher‐order interactions, were simplified by sequentially eliminating nonsignificant terms and interactions to establish a minimal model (Crawley 2012). The significance of the explanatory variables was established using either a likelihood ratio test (which is approximately distributed as a Chi‐square distribution, Bolker 2008) or an *F* test. The significant Chi‐square or *F* values given in the text are for the minimal model, whereas nonsignificant values correspond to those obtained before the deletion of the variable from the model. A posteriori contrasts were carried out by aggregating factor levels together and by testing the fit of the simplified model using an LRT (Crawley 2012).

To analyze the existence of a circadian rhythm in the parasite dynamics during the acute stage of infection, we used a method for identifying periodicity in large‐scale biological rhythm that allows us to account for the between‐day variation of parasitemia (see Supporting Information Materials, **S1 Text**). Briefly, to correct for non‐stationarities in the host parasitamia caused by the dramatic changes in parasitemia during the acute phase of the infection (Figure 1), we first detrended the signal by subtracting from each data point an estimate of the slow‐timescale dynamics of the parasitemia. We then estimated the frequency content of these stationary parasitemia signals and we used the Lomb–Scargle method to assess the statistical significance of the frequency distribution. We also looked for an effect of the time of the day on this detrended signal using a mixed‐effect model. We provide a link to a github notebook with a step‐by‐step description of this procedure and a code that may be used to analyze other within‐host time series (https://github.com/QCaudron/timing_malaria_transmission).

## Results

### THEORY: EVOLUTION OF ADAPTIVE RHYTHMICITY

Our analysis reveals that the evolution of the timing of pathogen transmission can be understood in the classical theoretical framework of virulence evolution. The invasion condition for a mutant pathogen is particularly useful if one examines the dynamics of a mutant with a time‐varying transmission strategy in a resident pathogen population with a strategy that does not vary with time (i.e., β_1_ is constant and $cov_{t}\left(A,B_{1}\right)=0$). The mutant will invade only if $s_{M}>0$, which yields:

(6) $$cov_{t}\left(A,B_{1M}\right)>\tilde{A}\left(\frac{\beta_{1}}{\left(d+\alpha \right)}-{\tilde{B}}_{1M}\right).$$

This condition means that the mutant will invade if the covariance between its time‐varying transmission strategy and mosquito activity is larger than the cost of virulence. Another way to express this condition is illustrated in Figure 2 in which the different panels represent different pathogen strategies (different patterns of fluctuation of parasitemia, in red) in a fluctuating environment characterized by periodic fluctuation in vector activity (in black and gray). A higher investment in transmission is beneficial if the vectors are available or active (i.e., when there is an overlap between red and gray shades). In contrast, a higher investment in transmission is costly if no vectors are available (higher investment in transmission may kill the host and reduce the duration of infection). Pathogens in Figure 2A and B adopt constant transmission strategies and the outcome of the competition between these two strategies depend only on the cost of virulence (the right hand side of equation (6)). The strategy that maximizes $\frac{\beta_{1}}{\left(d+\alpha \right)}$ is evolutionary stable, and will outcompete all other constant transmission strategies. A time‐varying strategy (Fig. 2C and D), however, may be a way to reduce the cost of virulence when mosquitoes are not available. A competitive time‐varying strategy is a strategy in which the covariance, $cov_{t}\left(A,B_{1M}\right)$, between mosquito activity and investment in transmission is large (i.e., strategy C is better than strategy D). In other words, this temporal covariance is a measure of the adaptive nature of time‐varying transmission.

Our theoretical analysis focuses on the evolution of a constitutive time‐varying investment in transmission to match a fast and periodic fluctuation of vector activity. But when the fluctuation of the environment is slower and/or is less predictable, it may be more adaptive to monitor environmental changes and to induce phenotypic modifications accordingly (e.g., Kussell & Leibler 2005, Greischar et al. 2016). In malaria, we developed a similar argument to analyze the evolution of inducible investment in transmission after mosquito bites (Cornet et al. 2014).

### EXPERIMENT: “HAWKING HYPOTHESIS” IN AVIAN MALARIA

**Figure 3:**
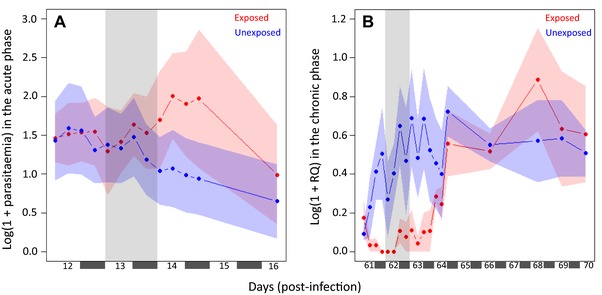
Parasitemia increases upon exposure to mosquito bites. The dynamics in birds exposed or unexposed to mosquito bites is represented in red and blue, respectively. Mosquito exposure took place **(A)** day 13 (at 06:00, 12:00, 18:00, and 00:00 h, parasitemia measured using blood smear counts) and **(B)** day 62 (at 06:00, 12:00, 18:00, and 00:00 h, parasitemia measured by qPCR) post‐infection.

Blood parasitemia initially followed a bell‐shape function typical of acute *Plasmodium* infections: peaking at day 12 post‐infection and decreasing thereafter (Fig. 1). The infection subsequently entered a long‐lasting chronic state, which was characterized by a low blood parasitemia over several weeks (Fig. 1). During the acute phase of the infection, and before the exposure to the mosquitoes, there was no significant difference in the parasitemia of the hosts assigned to the “exposed” and “unexposed” treatments (model 1: χ²1 = 0.01, *P* = 0.941, Fig. 3A). However, after they had been exposed to the mosquito bites, the acute‐phase parasitemia of the “exposed” birds was significantly higher than that of their “unexposed” counterparts (model 2: χ²_1_ = 8.59, *P* = 0.003, Fig. 3A). This effect was short‐lived and only lasted around 48 h (peak reached in 24 h**,** Fig. 3A). During the chronic phase of the infection, there was a significant difference in the parasitemia of the birds before the exposure session (model 3: χ²_1_ = 10.83, *P* = 0.001, Fig. 3B): “unexposed” birds had a higher parasitemia than “exposed” hosts. After exposure to mosquito bites, while the parasitemia of the “unexposed” birds did not vary (model 4: χ²_1_ = 0.086 p = 0.771, Figure 3B), the parasitemia of the “exposed” chronically‐infected hosts increased over time (peak reached in 6 days, model 5: χ²_1_ = 22.99, *P* < 0.0001, Fig. 3B).

### DAILY FLUCTUATIONS OF BLOOD PARASITAEMIA

The periodicity of the fluctuations in bird parasitemia was explored using a statistical approach that takes into account the overall within‐host dynamics of *Plasmodium* infection during the acute phase of the infection (see Supporting Information, **S1 Text**). In spite of a limited number of samples, this analysis suggests that bird parasitemia fluctuates periodically with a peak in the late afternoon (see Supporting Information). Another way to explore fluctuations of parasitemia throughout the day was to test the effect of time of day (06:00, 12:00, 18:00, and 00:00 h) on detrended parasitemia in the acute stage of infection and on parasitemia in the chronic stage of infection. To avoid the potential confounding effect of mosquito bites on parasite dynamics (Fig. 3) only “unexposed” birds were included in this analysis. We found an effect of the time of day on bird parasitemia in both the acute and the chronic stages of infection (model 6: χ²_1_ = 10.807, *P* = 0.013, model 7: χ²_1_ = 8.831, *P* = 0.032, respectively). In both stages parasitemia was higher in the evening than in the morning (Fig. S1).

**Figure 4:**
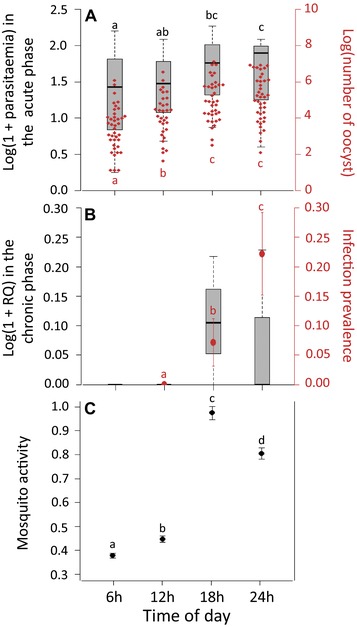
Avian malaria parasite times its within‐host dynamics to match mosquito activity. (A) Daily fluctuations of *Plasmodium* transmission in the acute phase of the infection (session 1: day 13 post‐infection, see Fig. 1). Boxplot represent the blood parasitemia (Log(1 + parasitemia)) of the exposed birds measured using blood smear counts at 06:00, 12:00, 18:00 and 00:00 h, 13 days after the infection by *Plasmodium*. The red points represent the distribution of the number of oocysts in the midgut of *Plasmodium*‐infected females 7 days after the blood meal. **(B)** Daily fluctuations of *Plasmodium* transmission in the chronic phase of the infection (session 2: day 62 post‐infection, see Fig. 1). Boxplot represent the blood parasitemia (Log(1 + relative quantification values)) of the exposed birds measured by qPCR at 06:00, 12:00, 18:00, and 00:00 h, 62 days after the infection by *Plasmodium*. The red points represent the prevalence of *Plasmodium* infection in females 7 days after the blood meal (proportion of blood‐fed mosquitoes with at least one oocyst). **(C)** Daily fluctuations of mosquito activity. The figure shows the constant hazard rate (mosquito blood feeding rate per hour) and the standard error estimated from the survival analyses (see Materials and Methods) for each treatment (time of day). A higher hazard rate equates to higher mosquito activity. Levels not connected by same letter are significantly different.

To examine the consequences of daily fluctuation of parasitaemia on parasite transmission in both the acute and chronic phases of the infection, we then focused our analyses on “exposed” birds. In the acute phase of the infection, we found a significant effect of the time of day on blood parasitemia (model 8: χ²_1_ = 11.58, *P* = 0.009, Fig. 4A). The parasitemia was highest at 18:00 and 00:00 h and lowest early in the morning (06:00 h, Fig. 4A). During the chronic phase of the infection blood parasitemia was very low in all exposed birds (parasitemia <0.001%, Fig. 1). Molecular methods, however, allowed us to detect daily variations in parasitemia. Parasite burden was null at 06:00 and 12:00 h, or below the detection levels, but increased in the evening (Fig. 4B).

#### Daily fluctuations of *Plasmodium* transmission

As mentioned above, the aim of this section was to investigate whether fluctuations in blood parasitemia translate into fluctuations in transmission to mosquitoes. For this purpose, we first quantify the number of oocysts in mosquitoes fed at different times of the day. We then explore whether these differences can be explained by differences in the amount of parasites ingested by the mosquitoes at different times of the day. Finally, we explore whether the fluctuations in blood parasitemia and mosquito infectivity match the daily patterns of mosquito activity.

In the acute stage, the mosquito infection prevalence was 100% for all feeding times. Blood feeding time, however, had a very significant effect on the oocyst burden of mosquitoes (model 9: χ²_1_ = 42.69, *P* < 0.0001, Fig. 4A). Females that fed in the evening (18:00 and 00:00 h) had more than twice as many oocysts as those feeding at noon (contrast analyses: 12:00/18:00 h: χ²_1_ = 8.28, *P* = 0.004, 12:00/00:00 h: χ²_1_ = 13.92, *P* < 0.0001, oocysts burden: 12:00 h: mean ± SE: 108 ± 25; 18:00 h: 262 ± 51; and 00:00 h: 314 ± 52) and noon‐feeding mosquitoes had significantly more oocysts than those feeding in the early morning (contrast analyses: 06:00/12:00 h: χ²_1_ = 5.03, *P* = 0.025, oocysts burden: 06:00 h: 62 ± 14). As expected, hematin, a proxy for blood meal size, has an impact on mosquito oocyst burden (model 9: χ²_1_ = 49.17, *P* < 0.0001). Crucially, however, the time of day has no impact on hematin production (model 10: χ²_1_ = 6.54, *P* = 0.091) implying that the blood meal sizes do not change according to the feeding times. An impact of bird parasitemia on oocyst burden was observed but only when the feeding time was removed from our statistical model (model 9: with time of day as covariate χ²_1_ = 1.70, *P* = 0.192, model 11: without time of day as covariate χ²_1_ = 15.09, *P* < 0.001).

The quantification of parasites ingested by mosquitoes showed a significant positive correlation with both hematin and time of day (model 12: χ²_1_ = 28.01, *P* < 0.0001, χ²_1_ = 41.71, *P* < 0.0001, respectively). The quantity of parasite ingested by mosquito was highest at midnight (00:00 h) and lowest early in the morning (06:00 h). Bird parasitemia also had an impact on the quantity of parasites ingested by females but only when the time of day was removed from the statistical model (model 12: with time of day as covariate χ²_1_ = 0.12, *P* = 0.727, model 13: without time of day as covariate χ²_1_ = 3.62, *P* = 0.047).

In the chronic stage of the infection, mosquito infection prevalence varied throughout the day (model 14: χ²_1_ = 6.98, *P* = 0.030, Fig. 4B). Infection prevalence was 0% at noon, 7% (mean ± s.e: 7.1 ± 4) at 18:00 h, and 22% (22.2 ± 7.1) at 00:00 h (no data available for 06:00 h, contrast analyses: 12:00/18:00 h: χ²_1_ = 3.89, *P* = 0.049, 12:00/00:00 h: χ²_1_ = 6.34, *P* = 0.012, 18:00/00:00 h: χ²_1_ = 3.91, *P* = 0.048). However, oocyst numbers were too low (all infected females had a single oocyst) to detect any effect of time of day on parasite burden. Bird parasitemia and blood meal size (hematin) had no impact on mosquito infection prevalence (model 14: with time of day as covariate χ²_1_ = 0.58, *P* = 0.447, model 15: without time of day as covariate χ²_1_ = 1.64, *P* = 0.201, model 14: with time of day as covariate χ²_1_ = 0.64, *P* = 0.725, model 15: without time of day as covariate χ²_1_ = 0.17, *P* = 0.679, respectively).

### DAILY FLUCTUATIONS OF MOSQUITO ACTIVITY

**Figure 5:**
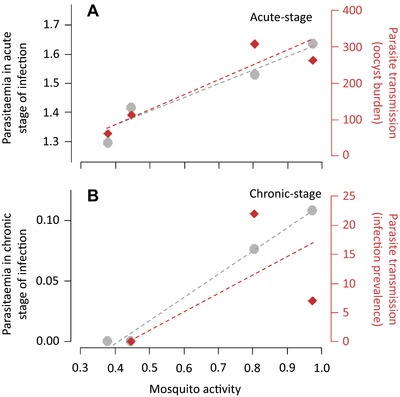
Validation of the “Hawking hypothesis” in the avian malaria system. (A) Correlation between mosquito activity (constant hazard rate estimated from model 14, Table S1) and both bird parasitemia quantified using blood smear counts (log(1 + parasitemia), in grey) and parasite transmission to mosquito (oocyst burden, in red) in the acute stage of the infection. (B) Correlation between mosquito activity (constant hazard rate estimated from model 16, **TableS1**) and bird parasitemia quantified using qPCR (log(1 + relative quantification values), in grey) and parasite transmission to mosquito (infection prevalence (%), in red) in the chronic stage of the infection.

Mosquito activity did not depend on whether they fed on birds in the acute or chronic stages of infection (model 16: χ²_1_ = 2.229, *P* = 0.135, Fig. 4C). However, mosquito activity varied significantly with the time of day (model 16: χ²_1_ = 204.15, *P* < 0.0001, Fig. 4C). Overall, the activity of vectors was higher in the evening (18:00 h, 00:00 h) than in the morning (06:00 and 12:00 h). The maximal activity was observed at dusk (contrast analyses: 18:00/00:00 h: χ²_1_ = 28.78, *P* < 0.0001, 18:00/12:00 h: χ²_1_ = 148.89, *P* < 0.0001, 18:00/06:00 h: χ²_1_ = 166.48, *P* < 0.0001, Fig. 4C) and the minimal activity at dawn (contrast analyses: 06:00/12:00 h: χ²_1_ = 4.09, *P* = 0.026, 06:00/00:00 h: χ²_1_ = 38.90, *P* < 0.0001, Fig. 4C). Interestingly, these daily variations in mosquito activity were positively correlated with bird parasitemia and parasite transmission to mosquito in both the acute and the chronic stages of the infection (Fig. 5).

## Discussion

Temporal fluctuations of the activity of mosquito vectors have profound consequences on malaria transmission (Barrozo et al. 2004; Lalubin et al. 2013). Here, we provide evidence that *Plasmodium* parasites have evolved two different and complementary transmission strategies to cope with these variations of their environment: a constitutive time‐varying strategy that generates a covariance between parasite investment in transmission and vector activity and a plastic, fast acting strategy that allows the parasite to react rapidly to the presence of mosquitoes.

First, our theoretical model indicates that fast and predictable oscillations in mosquito activity can select for a constitutive time‐varying strategy in the parasite, provided this strategy generates a positive covariance between the activity of the vector and the parasite's investment in transmission (see equation (6)). Our experimental results show both that the activity of *Culex* mosquitoes oscillates throughout the day in a predictable way (Fig. 4C) but also, that these daily fluctuations of mosquito activity are matched with periodic fluctuations in malaria transmission during both phases of the infection (i.e., acute and chronic, Fig. 5). This positive covariance supports the “Hawking hypothesis” and the idea that this time‐varying transmission may result from an adaptation of the pathogen.

Second, our experiment demonstrates the existence of plastic transmission strategies enabling avian malaria parasites to respond to mosquito bites. In a previous study, we showed that mosquito bites stimulate within‐host growth and investment in transmission during the chronic phase of *Plasmodium relictum* infections (Cornet et al. 2014). In the present study, we obtain a similar effect in the chronic but also in the acute phase of the infection. This plastic transmission strategy is expected to evolve when variations in the abundance of their mosquito vectors are less predictable (Cornet et al. 2014; Reece & Mideo 2014). During the chronic phase of the infection, such plastic transmission strategies may allow the parasite to react to the seasonal variations in mosquito abundance and to reactivate its transmission when mosquitoes are around (Cornet et al. 2014; Reece & Mideo 2014; Gandon 2016). During the acute phase of the infection, this strategy may also allow the parasite to respond to unexpected variations in the abundance of mosquitoes driven by stochastic processes such as variations in temperature and humidity (Yamana & Eltahir 2013). Interestingly, we also found that exposure to mosquito bites in the acute phase of the infection has a long‐term negative impact on the parasitemia in the chronic stage of the infection (before the exposure to the second batch of mosquito bites). We do not have a good explanation for this long‐term effect of mosquito bites. It is tempting to speculate that mosquito bites may have the potential to trigger a higher bird immune response (as demonstrated by Donovan et al. (2007) in rodent malaria) that could result in a lower parasitemia during the chronic phase. However, a demonstration of an effect of mosquito bites on bird immunity remains to be carried out in avian malaria.

In spite of the match between these theoretical predictions and our experimental results, our adaptive hypothesis is challenged by alternative explanations for the existence of periodic variations in parasitemia and mosquito infection. Several studies suggest that the dynamics of the infectivity of *Plasmodium* might not be underpinned by the feeding activity cycle of its vector but induced by the vertebrate immunity (see Mideo et al. 2013), whose activity is known to vary during the day (Scheiermann et al. 2013; Curtis et al. 2014). This variation may alter the number and/or the infectiousness of gametocytes and explain (at least partly) the increase of transmissibility during the evening. It would be interesting to monitor whether the efficacy of the birds' immune system to fight against a *Plasmodium* infection fluctuates throughout the day, and to evaluate its potential effect on the transmissibility of avian malaria.

In addition, the increase in mosquito infection may also be explained by physiological cycles in the vector. Daily cycles in the production of immune compounds (Rund et al. 2016; Tsoumtsa et al. 2016) or molecules (e.g., nutrients) used by *Plasmodium* (Carter et al. 2007; Dinglasan et al. 2007) may impact the viability of ookinetes or their ability to invade the midgut epithelia. One way to quantify this effect would be to perform similar experiments with vectors with the circadian rhythm experimentally inversed (jet‐lagged). Reversed patterns of time‐varying infectivity in jet‐lagged and control mosquitoes would demonstrate the importance of the effect of the circadian rhythm of the insect vector. In contrast, if both jet‐lagged and control mosquitoes exhibit similar patterns of infection, this would indicate that the infectivity is under the parasite's control and would support the “Hawking hypothesis”.

The most efficient way to demonstrate unequivocally the adaptive nature of these time‐varying transmission strategies, however, would be to perform experimental evolution (Johnson 2005). For instance, does the parasite lose its ability to react to mosquito bites if the parasite is always transmitted from bird to bird by intraperitoneal injection (Pigeault et al. 2015)? Could the parasite evolve other patterns of daily investment in transmission if the mosquitoes are allowed to feed on birds at very specific time of the day? Avian malaria provides a perfect experimental system to carry out such experiments. Earlier studies have observed a great degree of variation in the period and in the phase of the fluctuations of within‐bird dynamics. For instance, *P. circumflexum* has a periodicity of 48 h peaking in the late afternoon, while *P. elongatum*’s periodicity is 24 h and peaks in the early morning (see Hewitt 1940 for a review). Besides, the amplitude of the fluctuations of parasitemia reported in some of these earlier experimental studies is orders of magnitude higher than the one we observed in the present study (Taliaferro 1925, Huff & Bloom 1935, Hewitt 1940). What factors explain the maintenance of such a large amount of natural variation? Additional experimental studies using different avian *Plasmodium* lineages would yield unique perspectives on the adaptive nature of the rhythmicity of malaria within‐host dynamics. The genomic analysis of evolved lines would also yield new candidate genes governing these key adaptations. This deeper understanding of malaria transmission may thus yield practical implications for the control of human malaria parasites.

Associate Editor: Prof. J. Slate

## Supporting information


**Figure S1**. Daily fluctuations of parasitaemia in unexposed birds.

**Table S1**. Description of statistical models used.

**Text S1**. Daily fluctuations of parasitaemia: a new methodology.
