# Supplementary material for: Timing malaria transmission with mosquito fluctuations
Source: Evol Lett. 2018 Jun 22;2(4):378–89. doi: 10.1002/evl3.61 (PMC6122125; doi:10.1002/evl3.61)
Supplement: Supplementary file 1 — Figure S1. Daily fluctuations of parasitaemia in unexposed birds. [file EVL3-2-378-s001.pdf]

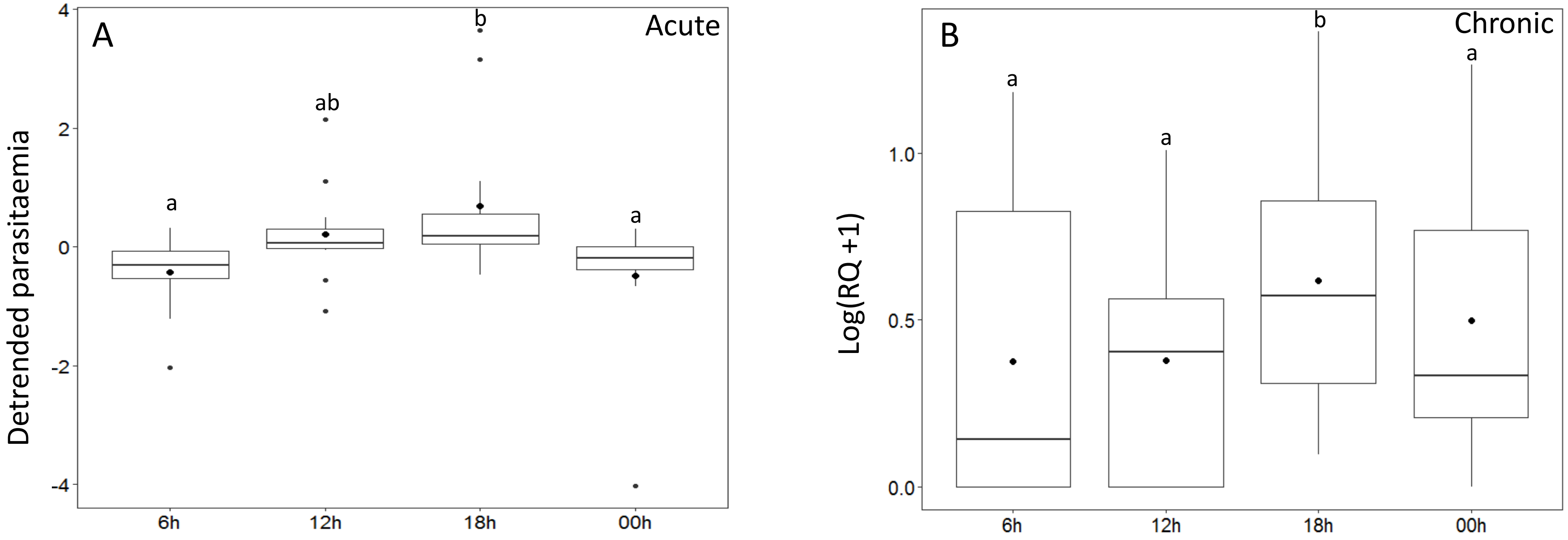

Figure S1: Daily fluctuations of parasitaemia in unexposed birds. (A) Daily fluctuations of parasitaemia in acute phase of infection. Boxplot represent the detrended parasitaemia (See Supporting Information) of the unexposed birds measured using blood smear counts at 6h, 12h, 18h and 00h, days 12, 13 and 14 after the infection by *Plasmodium*. (B) Daily fluctuations of parasitaemia in chronic phase of infection. Boxplot represent the parasitaemia (Log RQ+1) of the unexposed birds measured by qPCR at 6h, 12h, 18h and 00h, days 61, 62, 63 and 64 after the infection by *Plasmodium*.
