# Supplementary material for: Timing malaria transmission with mosquito fluctuations
Source: Evol Lett. 2018 Jun 22;2(4):378–89. doi: 10.1002/evl3.61 (PMC6122125; doi:10.1002/evl3.61)
Supplement: Supplementary file 2 — Table S1. Description of statistical models used. [file EVL3-2-378-s002.docx]

**Timing malaria transmission with mosquito fluctuations**

Romain Pigeault ^1,2^, Quentin Caudron^3^, Antoine Nicot^4^, Ana Rivero^1^, Sylvain Gandon^4^

**S1 Table: Description of statistical models used**

**Table S1**: **Description of statistical models used.** N gives the number of birds or mosquitoes included in each analysis. "Maximal model" gives the complete set of explanatory variables included in the model. "Minimal model" gives the model containing only the significant variables and their interactions. Parentheses indicate variables fitted as random factors. Square brackets indicate the error structure used (n: normal errors, b: binomial errors). Acronyms: Par = parasitaemia, exposure = expose or not to mosquito bites, day = day post-infection, DtrdPar = detrended parasitaemia, RQ = relative quantification values, Oo = oocyst burden, prev = infection prevalence, Activity = time required to take a blood meal, experiment = acute and chronic experimental session.

| **Variable of interest** | **Resp. variable** | **Model Nb.** | **N** | **Maximal model** | **Minimal model** | **Rsubroutine [err struct.]** |
| --- | --- | --- | --- | --- | --- | --- |
| **Effect of mosquito bites** |  |  |  |  |  |  |
| Parasitaemia (days 12-13) | log(1+Par) | 1 | 32 | exposure*time+(1/Bird) | 1+(1/Bird) | lme[n] |
| Parasitaemia (days 13-16) | log(1+Par) | 2 | 72 | exposure*time+(1/Bird) | exposure*time+(1/Bird) | lme[n] |
| Parasitaemia (days 61-62) | log(1+RQ) | 3 | 28 | exposure*time+(1/Bird) | exposure*time+(1/Bird) | lme[n] |
| Parasitaemia (days 62-70, unexposed) | log(1+RQ) | 4 | 40 | time+(1/Bird) | 1+(1/Bird) | lme[n] |
| Parasitaemia (days 62-70, exposed) | log(1+RQ) | 5 | 30 | time+(1/Bird) | time+(1/Bird) | lme[n] |
| **Daily fluctuation of parasitaemia** |  |  |  |  |  |  |
| Parasitaemia (day 12, 13, 14, unexposed) | DtrdPar | 6 | 48 | time*day+(1/Bird) | time+(1/Bird) | lme[n] |
| Parasitaemia (day 61, 62, 63, 64, unexposed) | log(1+RQ) | 7 | 60 | time*day +(1/Bird) | Time+day +(1/Bird) | lme[n] |
| Parasitaemia (day 13, exposed) | log(1+Par) | 8 | 12 | time+(1/Bird) | time+(1/Bird) | lme[n] |
| **Daily fluctuation of transmission** |  |  |  |  |  |  |
| Oocyst (acute) | log(Oo) | 9 | 144 | Par*time*hm+(1/Bird) | time+hm+(1/Bird) | lme[n] |
| Haematin | hm | 10 | 144 | time+(1/Bird) | 1+(1/Bird) | lme[n] |
| Oocyst (acute) | log(Oo) | 11 | 144 | Par*hm+(1/Bird) | Par+hm+(1/Bird) | lme[n] |
| Parasite in mosquito (acute) | log(1+RQ) | 12 | 175 | Par*time*hm+(1/Bird) | time+hm+(1/Bird) | lme[n] |
| Parasite in mosquito (acute) | log(1+RQ) | 13 | 175 | Par*hm+(1/Bird) | Par+hm+(1/Bird) | lme[n] |
| Prevalence (chronic) | prev | 14 | 91 | Par*time*hm+(1/Bird) | time+(1/Bird) | lmer[b] |
| Prevalence (chronic) | prev | 15 | 91 | Par*hm+(1/Bird) | time+(1/Bird) | lmer[b] |
| **Daily fluctuation of mosquito activity** |  |  |  |  |  |  |
| Mosquito activity | activity | 16 | 1125 | time+experiment+(1/Bird) | time+(1/Bird) | survreg[exp] |
