# Supplementary material for: Timing malaria transmission with mosquito fluctuations
Source: Evol Lett. 2018 Jun 22;2(4):378–89. doi: 10.1002/evl3.61 (PMC6122125; doi:10.1002/evl3.61)
Supplement: Supplementary file 3 — Text S1. Daily fluctuations of parasitaemia: a new methodology. [file EVL3-2-378-s003.docx]

**Timing malaria transmission with mosquito fluctuations**

Pigeault, R., Caudron, Q., Nicot, A., Rivero, A., Gandon, S.

**S1 Text: Daily fluctuations of parasitaemia: a new methodology**

We propose a new methodology allowing to correct for non-stationarities in the host parasitaemia. We provide a link to a github notebook with a step-by-step description of the procedure presented in the supplement and a code that may be used to analyse other within-host time series (<https://github.com/QCaudron/timing_malaria_transmission>).

**Overview**

The goal of this analysis is to explore periodicity in the host parasitaemia, specifically to search for circadian frequencies. First, we correct for non-stationarities in the host parasitaemia caused by the large-scale changes in within-host dynamics during the acute phase of the infection. Then, we estimate the frequency content of the parasitaemia signals and assess the statistical significance of the frequency distribution. Results show some evidence of possible circadian or quasicircadian frequencies in parasitaemia.

**The dataset**

The time-series for host parasitaemia are short, being composed of only twelve datapoints captured at six-hour intervals over three days. In addition, measurements are noisy: with three samples per point in time, mean coefficients of variation range from 0.057 to 0.324 per bird. We begin by taking the mean of these samples for each host in order to reduce noise. On certain hosts, we note a strong nonstationarity, driven by the within-host infection dynamics (**Fig. S1**).

Figure S1. Parasitaemia values (scatter), with mean (line) and standard error (fill) for the control hosts. Grey shadows represent night (18:00 to 06:00).

**Detrending**

In the acute phase of the infection, significant trends in parasitaemia may be present in the signals, although at slower timescales than the circadian rhythms (see **Fig. 1** in the main text) that would be present, should the parasite be able to invest in transmission synchronously with the daily cycles of its vector’s activity. We detrend the time-series by subtracting an estimate of the slow-timescale dynamics of the parasitaemia signals, leaving behind a zero-mean, stationary signal that can then undergo spectral analysis. These dynamics are estimated using Gaussian process [1] regression using a radial basis function covariance kernel. The result can be seen in **Fig. S2**, which shows a smooth, continuous function estimated from the parasitaemia of each host. The mean of the detrended signal no longer varies with time; this is an assumption underlying the subsequent spectral analysis. Gaussian process regression was performed in Python with the scikit-learn package [2].

Figure S2. Left: mean parasitaemia (blue); estimated slow-changing acute infection dynamics, as found by Gaussian process regression (red). Right: the detrended parasitaemia, found by subtracting the estimated slow-timescale dynamics from the mean parasitaemia. Grey shadows represent night (18:00 to 06:00).

**Frequency analysis**

The goal of spectral analysis is to break down a signal into its component frequencies. All signals can be represented as a (potentially infinite, potentially time-dependent) sum of sinusoids with varying frequencies, phases, and amplitudes. When looking for repeating patterns in a signal, such as circadian dynamics, considering the signal in its frequency space can provide evidence of its presence or absence. The periodogram, an estimate of a signal's spectral density, describes the power of the frequency components that make up the signal.

The Lomb-Scargle method [3], also known as least-squares spectral analysis, can estimate the periodogram of signals with heteroscedastic errors. Due to the nonstationary nature of the original signals, where acute phase dynamics may have resulted in significant variation of the mean over time, errors cannot be assumed to be homoscedastic in the detrended signals. Furthermore, the Lomb-Scargle method naturally enables a significance threshold [4] to be found beyond which any peaks in the frequency spectrum can be said to be statistically significant. Lomb-Scargle periodogram estimation and significance bootstrapping were performed in Python using the astroML package [5].

Figure S3. Left: the distribution of the frequency content in the detrended parasitaemia; the black line shows the 95% confidence limit as found by bootstrapping. Right: the detrended signal (blue), overlaid with the circadian sinusoid that best fits it (red).

**The phase of the circadian frequency**

The analysis presented in **Fig. S3** shows some evidence for a peak in the periodogram around the daily frequency (that is, a circadian rhythm). By considering the detrended parasitaemia of Unexposed Host #2 (in which the circadian dynamic was found statistically significant), and performing a least-squares fit of a sinusoid with a daily frequency (as shown in **Fig. S3**), we find that the phase of this daily dynamic occurs in the late afternoons (16:03), which coincides with periods of high mosquito activity (see also **Fig. 3** in the main text).

# Bibliography

1. Williams CE. 2006 *Gaussian Processes for Machine Learning.* MIT Press

2. Pedregosa F, Varoquaux G, Gramfort A, Michel V, Thirion B, Grisel O, et al. 2012 Scikit-learn: machine learning in python. *JMLR*. **12,** 2825-2830. (doi: 10.1145/1961189.1961199)

3. Zechmeister M, & Kürster M. 2009 The generalised lomb-scargle periodogram. *A & A.* **496**, 577–584. (doi: 10.1051/0004-6361:200811296)

4. Scargle J. 1982 Studies in astronomical time series analysis. II. Statistical aspects of spectral analysis of unevenly spaced data. *Astrophys Journ.* **293,** 835-853. (doi: 10.1086/160554)

5. VanderPlas J, Connolly A, Ivezić Ž, Gray A. 2012 Introduction to astroML: Machine learning for astrophysics. *Proceedings of the Conference on Intelligent Data Understanding (CIDU)*. 47-54. (doi:10.1109/CIDU.2012.6382200)
